# *PySOFI*: an open source Python package for SOFI

**DOI:** 10.1016/j.bpr.2022.100052

**Published:** 2022-03-30

**Authors:** Yuting Miao, Shimon Weiss, Xiyu Yi

**Affiliations:** 1 Department of Chemistry and Biochemistry, University of California, Los Angeles California; 2 Department of Physiology, University of California, Los Angeles California; 3 Department of Physics, Institute for Nanotechnology and Advanced Materials, Bar-Ilan University, Ramat-Gan, Israel; 4 Lawrence Livermore National Laboratory, Livermore, California

## Abstract

Super-resolution optical fluctuation imaging (SOFI) is a highly democratizable technique that provides optical super-resolution without requirement of sophisticated imaging instruments. Easy-to-use open-source packages for SOFI are important to support the utilization and community adoption of the SOFI method, they also encourage the participation and further development of SOFI by new investigators. In this work, we developed *PySOFI*, an open-source Python package for SOFI analysis that offers the flexibility to inspect, test, modify, improve, and extend the algorithm. We provide complete documentation for the package and a collection of Jupyter Notebooks to demonstrate the usage of the package. We discuss the architecture of *PySOFI* and illustrate how to use each functional module. A demonstration on how to extend the *PySOFI* package with additional modules is also included in the *PySOFI* package. We expect *PySOFI* to facilitate efficient adoption, testing, modification, dissemination, and prototyping of new SOFI-relevant algorithms.

## Why it matters

Super-resolution optical fluctuation imaging (SOFI) is a highly democratizible technique for optical super-resolution without requirement for sophisticated imaging instruments. In this work, we developed PySOFI, an open-source Python package for SOFI analysis. We also provide complete documentation for the package and a collection of Jupyter Notebooks to demonstrate the usage of the package. We expect PySOFI to facilitate efficient adoption, testing, modification, dissemination, and prototyping of new SOFI-relevant algorithms.

## Introduction

Super-resolution optical fluctuation imaging (SOFI) (1) is a widely used optical super-resolution method applicable for a broad range of conditions, where sophisticated controls on the instrument and sample preparations are not required. It has attracted an active community of practitioners over a decade. The advancements utilizing this technology include innovations in blinking dyes and fluorescent proteins, sample preparation (2, 3, 4, 5, 6), illumination schemes, experiment designs, data processing methods (7, 8, 9, 10, 11, 12, 13, 14, 15), and integration with other methods (16, 17, 18, 19, 20, 21, 22, 23).

SOFI is compatible with simple wide-field imaging systems to process image stacks of samples that exhibit fluctuation of optical signals. It is assumed that the position of the signal sources are static over the time course of acquisition, and the optical fluctuations can be induced by either the stochastic blinking of the fluorophores, the diffusion and stochastic binding of fluorophores to static binding sites, or the fluctuation of optical scatters (24,25).

Various prior studies have provided detailed explanations on the SOFI principles (1,8,7,24,26), and the current status of SOFI investigations is well summarized in a recent review article by Pawlowska et al. (27). Interested readers are directed to reference (28) for insights about moments, cumulants, and their interplay. Here, we provide a brief review on the theory of SOFI processing. Given a sample with *N* emitters that blink independently with binary fluorescence intensity profiles constituting a fluorescence “on” state and a fluorescence “off” state, the fluorescence signal captured at a camera pixel located at $\vec{r}$ and time *t* is

(1) $$F\left(\vec{r},t\right)=\overset{N}{\sum_{k=1}}\varepsilon_{k}b_{k}\left(t\right)U\left(\vec{r}-\vec{r_{k}}\right),$$

where *k* is the emitter index, $\vec{r_{k}}$ is the location of the $k^{th}$ emitter, *U* is the point spread function (PSF) of the imaging system, $\varepsilon_{k}$ is the constant on-state brightness, and $b_{k}\left(t\right)$ is the time-dependent stochastic blinking profile. $b_{k}\left(t\right)$ equals 1 when the emitter is in the on state and 0 when the emitter is in the off state.

The first step of SOFI is to calculate the fluctuation of fluorescence signal around the temporal average for each pixel:

(2) $$\delta F\left(\vec{r},t\right)=F\left(\vec{r}\right)-\langle F\left(\vec{r}\right)\rangle_{t}=\overset{N}{\underset{k=1}{\sum }}\varepsilon_{k}\delta b_{k}\left(t\right)U\left(\vec{r}-\vec{r_{k}}\right).$$

Here, both $\varepsilon_{k}$ and *U* are constant, and only the fluctuation in the blinking profile $\delta b_{k}$ would affect δF. Then, the correlation functions can be calculated as follows:

(3) $$G_{n}\left(\vec{r_{1}},\vec{r_{2}},\cdots ,\vec{r_{n}};\tau_{1},\tau_{2},\cdots ,\tau_{n-1}\right)={\left\langle \delta F\left(\vec{r_{1}},t\right)\delta F\left(\vec{r_{2}},t+\tau_{1}\right)\cdots \delta F\left(\vec{r_{n}},t+\tau_{n-1}\right)\right\rangle }_{t}.$$

Eq. 3 represents the auto-correlation function when $\vec{r}=\vec{r_{1}}=\vec{r_{2}}=\cdots =\vec{r_{n}}$ and the cross-correlation function otherwise. Replacing $\delta F\left(\vec{r_{i}},\tau_{i-1}\right)$ with $\delta F_{i}$, Eq. 3 can be simplified as shown below:

(4) $$G_{n}\left(\delta F_{1},\delta F_{2},\cdots ,\delta F_{n}\right)=\left\langle \delta F_{1}\delta F_{2}\cdots \delta F_{n}\right\rangle .$$

Here $G_{n}\left(\delta F_{1},\delta F_{2},\cdots ,\delta F_{n}\right)$ indicates the joint moment of the set $\left\{\delta F_{i}|i\in \left[1,2,...,n\right]\right\}$. Next, the *n*th order joint cumulant of the set, $C_{n}\left(\delta F_{1},\delta F_{2},\cdots ,\delta F_{n}\right)$, can be derived from joint moments and joint cumulants of lower orders based on a recursive relation (see the Moment and cumulant reconstructions (E1) section). Note here that the joint moment and joint cumulant are generalized terms of correlation functions and cumulants, which are calculated from either auto-correlations or cross correlations with different choices of $\tau_{i}$ values and pixel combinations (11).

The *n*th-order cumulant functions can also be addressed based on fluorescence fluctuation of a multi-emitter system:

(5) $$C_{n}(\vec{r},\tau_{1},\tau_{2},\cdots ,\tau_{n-1})=\overset{N}{\sum_{k=1}}\varepsilon_{k}^{n}\omega_{n,k}(\tau_{1},\tau_{2},\cdots ,\tau_{n-1})U^{n}(\vec{r}-{\vec{r}}_{k}),$$

where $\omega_{n,k}(\tau_{1},\tau_{2},\cdots ,\tau_{n-1}))$ equals the *n*th-order cumulant of $\delta b_{k}\left(t\right)$. Detailed derivation can be found in (1,8,11,26). With *n*th-order cumulant analysis, the theoretical resolution improvement of SOFI is $\sqrt{n}$ fold. Such improvement increases to *n* when combined with deconvolution, presenting a great potential for further advancements for SOFI (1).

However, there are imperfections in high-order SOFI cumulants (e.g., cusp artifacts (26)), which can be explained under the framework of virtual emitter interpretation (26)); specifically, by comparing the similarity between Eqs. 1 and 5. The high-order SOFI cumulant image can be perceived as an image captured from a microscope with a PSF that is equivalent to the *n*th power of the original PSF (compare the terms that contain *U* between Eqs. 1 and 5), with emitters located at the same location as in the original sample but with emitter brightnesses replaced into $\varepsilon_{k}^{n}\omega_{n,k}$. Because $\omega_{n,k}$ is the *n*th-order cumulant of the blinking profile of the $k^{th}$ emitter, its value can be either positive or negative, which would introduce the cusp artifacts in the SOFI cumulant images when we display the absolute value of an image with adjacent positive and negative virtual emitters(26). We also demonstrated how the validity of one of the most widely used SOFI processing methods, bSOFI (7), is negatively impacted, but such findings have not received common awareness as of yet.

We believe an insightful and thorough understanding of the method is crucial to ensure solid advancements in both SOFI and SOFI-relevant innovations. However, for new investigators without prior experience with SOFI analysis, there is often a steep learning curve to fully understand, modify, and extend the existing open-source packages (7,29). The existing publicly available SOFI analysis routines are implemented in ImageJ (30), MATLAB (29,31), or Igor Pro (29). ImageJ requires professional programming skills if customization and modifications are required, while MATLAB and Igor Pro require paid licenses. Such limitations present a greater challenge for new investigators who are interested in joining the SOFI community but prefer not to use the existing packages blindly.

Here, we present *PySOFI*, an open-source package for SOFI analysis implemented in Python. Benefitting from the active open-source community and the abundance of free learning materials for Python, *PySOFI* offers an easy option for investigators interested in adopting the SOFI algorithm. *PySOFI* focuses on engaging the community and is designed to be simple, modular, and highly customizable. *PySOFI* is hosted on GitHub(32) to facilitate utilization, improvements, and continuous maintenance by interested users and developers. A collection of examples are provided in the form of Jupyter Notebooks. One can use *PySOFI* to explore and characterize SOFI analysis, validate the results from prior studies, and gain insights through exploration. *PySOFI* is also useful for the prototyping of new methods that extend the SOFI algorithm. Similar Jupyter Notebooks can be adapted to promote the new methods and improve the reproducibility of the results. We expect *PySOFI* to appeal to both beginners and experts, to facilitate innovations where modifications and extensions are required, and to further promote scientific advancements among scientists interested in SOFI.

The rest of the manuscript is organized as follows. We first provide an overview of the *PySOFI* package. Whe then provide discussions on the *PySOFI* software architecture design and analysis pipeline, together with analysis examples for various modules. In the end we summarize the work and discuss future directions.

### *PySOFI* overview

We designed a straightforward architecture for the *PySOFI* package. As shown in Table 1, *PySOFI* contains eight independent function modules (in the functions folder) and one data class (PysofiData). A detailed description of *PySOFI* is available in our online documentation(33). To get started with the installation, the user can follow the "Getting Started" page.

**Table 1:** *PySOFI* modules

Data class	pysofi.py	Defines the main data class called ”PysofiData”
Function	reconstruction.py	contains tools for cumulant and moment reconstruction
	finterp.py	contains tools for Fourier interpolation on ∗.tiff stacks for fSOFI processing
	filtering.py	contains tools for noise filtration along the time axis
	deconvsk.py	contains tools for shrinking kernel deconvolution
Modules	ldrc.py	contains tools for local dynamic range compression of images
	visualization.py	contains tools for visualization of the results
	moca.py	contains tools for multi-order cumulant analysis
	masks.py	contains tools for generating Gaussian kernels

*PySOFI* contains one data class and eight function modules. Detailed descriptions are available in the online documentation(33). Note that the moca.py and deconvsk.py modules involve non-peer-reviewed works and are beyond the scope of this work. This work focuses on the introduction of the software package *PySOFI,* therefore, moca.py and deconvsk.py are not discussed in this work.

**Figure 1:**
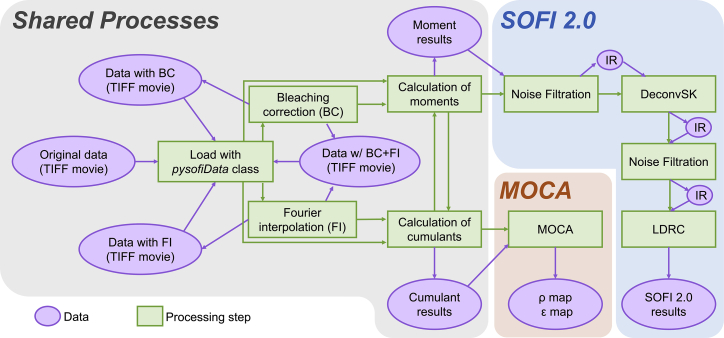
Data-flow diagram for *PySOFI*. Three collections of SOFI-analysis routines are implemented in *PySOFI*, as depicted in the diagram: Shared Processes, SOFI 2.0 analysis, and MOCA analysis. Green squares represent data-processing steps with functionalities labeled for each step. The purple ovals represent the data types as labeled in the diagram. IR represents intermediate results. The green arrow represents the direction of the data flow between different steps, and the purple oval represents input and output data types at different processing steps. Note that the multi-order cumulant analysis (MOCA) process and DeconvSK processing step involve non-peer-reviewed works, which are beyond the scope of this work and will not be discussed.

Fig. 1 provides the data-flow diagram that demonstrate the connections (arrows) between different processing steps (green squares) and different types of data (purple ovals). Three collections of SOFI analysis routines are implemented in the PysofiData class, including the “Shared Processes” that contain the traditional SOFI analysis steps (1) and the “SOFI 2.0” collection that contains the routines for SOFI 2.0 processing (11). In the Shared Processes block, the processing steps include bleaching correction (BC), Fourier interpolation (FI), and moment and cumulant calculations. The processing steps can be performed in various sequences (green arrows). In the SOFI 2.0 block, one can perform noise filtering and local dynamic range compression (*ldrc*) on the image. In the data-processing workflow, one can save and load the intermediate results (IR) for each processing step (purple arrows). For example, in the Shared Processes collection, the intermediate results (purple ovals) can be saved as separate new tiff files or stored as attributes in the PysofiData class object and then passed to another processing step (purple arrow).

The following modules are implemented to facilitate the *PySOFI* analysis pipeline. The reconstruction.py module provides capabilities for SOFI moments and cumulants calculations (1), as well as BC for a tiff movie. The finterp.py module provides FI on a tiff stack, which is used for FI stochastic optical fluctuation imaging (fSOFI) analysis (9). filtering.py and ldrc.py constitute a collection of modules relevant to SOFI 2.0 (11) analysis. Specifically, the filtering.py module is for pixel-wise noise filtering along the time axis, and the ldrc.py is for *ldrc* of images with a large dynamic range of pixel values (10). The masks.py module is used to generate Gaussian kernels, and the visualization.py module provides visualization options using an interactive visualization package Bokeh. The data-class (PysofiData) module is encapsulated in the pysofi.py file. The input parameters from the users, the raw data, and the intermediate results are bundled in the PysofiData object as attributes, and the processing steps as methods. The processing steps are implemented as function modules and imported and used in the data-class module. In summary, the specific functions are implemented in the function modules, while PysofiData serves the purpose of organizing the data-processing workflow.

In general, we adopted a simple architecture for *PySOFI* with a collection of independent function modules and only one class module (the data class). The functions are imported and used inside the data class across different methods as needed, and therefore the implementation is flexible with minimum repetition of codes. The function modules can be implemented, modified, and tested independently, ensuring flexibility and convenience for maintenance. Extending the package can be done by implementing additional function modules. It can be used as a standalone process or be integrated into the data-processing workflow through the PysofiData class. The investigators also have the flexibility to disseminate the *PySOFI* package and construct their own data-processing workflow (similar to the PysofiData class).

### Implementation of SOFI analysis using *PySOFI*

We provide a collection of Jupyter Notebooks (outlined in Table 2) as examples for *PySOFI* implementations and applications. The prefix (E#) of each filename is used as a reference to each notebook in the following text for simplicity. We present example *PySOFI* analysis steps (E1, E2, and E4–E6), a visualization of the result with a combined color map and a transparency map (E8), and the effect of data-acquisition length on SOFI reconstruction performance (E9 and E10). We also demonstrate SOFI 2.0 analysis (E11) and characterization of cusp artifacts (E12 and E13). Two processing steps (E3 and E7) address non-peer-reviewed methods that are beyond the scope of this work and therefore will not be discussed in this work. The analysis processes are integrated through the PysofiData class for all the notebooks except for the demonstration of noise filtration (E2).

**Table 2:** Jupyter notebook examples for *PySOFI*

Group	Notebook Name	Data	Section
Processing	E1_MomentCumulantReconstructions	Block1.tif (live-cell imaging)	Moment and cumulant reconstructions (E1)
	E2_NoiseFiltration	Block1.tif – Block20.tif (live-cell imaging)	Temporal Noise Filtering (E2)
	E3_ShrinkingKernelDeconvolution	Block10.tif (live-cell imaging)	N.A.
Steps	E4_LDRCMethod	Block1.tif (live-cell imaging)	ldrc (E4)
Demonstration	E5_FourierInterpolation	Block10.tif (live-cell imaging)	FI (E5)
	E6_BleachingCorrection	Bleach_SlowVaryingRho_frame2000_Emi51.tif (simulation)	BC (E6)
		3Emitters_frame5000_Emi3_close.tif (simulation)	N.A.
	E7_MOCA	SlowVaryingRho_frame2000_Emi51.tif (simulation)	N.A.
		RndomCurves_frame15000_rho04.tif (simulation)	
	E8_ResultVisualization	RndomCurves_frame15000_rho04.tif (simulation)	Result visualization (E8)
Analysis		frame1000_10000.npy	N.A.
Exploration	E9_ReconstructionConvergence	frame11000_15000.npy	N.A.
		frame16000_20000.npy (generated in E8)	
	E10_ReconstructionConvergence_SampleAnalysis	nobleach_frame20000_3.tif (simulation)	N.A.
SOFI 2.0 demonstration	E11_PysofiExample_LiveCellActinFilaments	Block1.tif - Block20.tif (live-cell imaging)	N.A.
Cusp artifacts	E12_CuspArtifactsDemo1_3Emitters	3Emitters_frame5000_Emi3_close.tif (simulation)	N.A.
Demonstrations	E13_CuspArtifactsDemo2_SlowVaryingRho	SlowVaryingRho_frame2000_Emi51.tif (simulation)	N.A.

We provide thirteen *PySOFI* demonstrations as Jupyter Notebooks, which can be categorized into to four groups (first column). The file name (second column) indicates the focus of each Jupyter Notebook. The relevant data sets (third column) are shared on figshare (34). Brief descriptions of most processing steps (E1, E2, E4, E5, and E6) and their notebooks are provided in the relevant section (fourth column). The theories behind E9 to E13 are not included in this work, but the relevant concepts are discussed in (26) and (11). The notebooks are the *PySOFI* implementations of the relevant methods to support the utilization of them. In particular, in E11, we show the general guidelines for performing SOFI 2.0 analysis on live-cell fluorescence-imaging results using *PySOFI*. Note that the multi-order cumulant analysis and shrinking kernel deconvolution processing steps involve non-peer-reviewed work, which is beyond the scope of this work; therefore, notebooks E3 and E7 are not discussed in this manuscript. N.A., not applicable.

In the text below, we provide brief descriptions of E1, E2, E4, E5, and E6. The complete detailed description and examples are provided in the Jupyter Notebooks in the online GitHub repository (32).

### Moment and cumulant reconstructions (E1)

Traditionally, SOFI achieves resolution enhancement by computing different orders of cumulants of optical-signal fluctuations in time. The theoretical resolution enhancement for SOFI is $\sqrt{n}$ fold for the *n*th-order SOFI cumulant. Once combined with deconvolution, the theoretical resolution enhancement can increase to n fold.

To obtain the *n*th order SOFI cumulant, one way is to construct the *n*th-order cumulant as a polynomial consists of moments from the first order to the *n*th order, as shown in the previous work (1). Another way, which is used by *PySOFI*, is to construct the following recursive relation: $Cum_{n}=G_{n}-\sum_{i=1}^{n-1}C_{n-1}^{i}\cdot Cum_{n-i}\cdot G_{i}$, where $Cum_{n}$ represents the *n*th-order cumulant, $G_{n}$ represents the *n*th-order-moment, and $C_{N}^{M}$ means the number of combinations of “N choose M.” Regarding the moment calculations, *PySOFI* support calculations of moments directly from the time series of each pixel. The moments can also be calculated as a reconstruction from a series of cumulants, as used in our previous study (11).

The calculation of cumulants and moments are the fundamental processing elements in the SOFI analysis. The PysofiData class organizes the analysis workflow and can be used to calculate both moments and cumulants. Essentially, the relevant function modules are imported and integrated in PysofiData to support such analysis. For example, the following scripts would calculate the fourth-order moment and cumulant of the specified tiff stack named *Block1.tif* through the PysofiData class:

# Load data into pysofidata object:

filepath = '../sampledata'

filename = 'block1.tif'

d = pysofi.pysofidata(filepath, filename)

# Calculate the fourth-order moment image:

m_im = d.moment_image(order=4)

# Calculate the fourth-order cumulant image:

k_set = d.cumulants_images(highest_order=4)

We can also directly import the function module, reconstruction.py to perform the relevant calculations. This option is designed to support the dissemination of the *PySOFI* package to facilitate independent analysis design, which is often useful when developing new methods built upon the SOFI analysis. The following scripts demonstrate how to perform such analysis with moment and cumulant calculations up to the fourth order:

# Import the relevant function modules and define the path and name for the data:

from pysofi import reconstruction as rec

filepath = '../sampledata'

filename = 'Block1.tif'

# Calculate the fourth-order moment image:

m_set = rec.calc_moments(filepath, filename, highest_order=4)

# Calculate the fourth-order cumulant image:

k_set = rec.calc_cumulants_from_moments(m_set)

More detailed demonstrations are available in the corresponding Jupyter Notebook (E1).

### Temporal noise filtering (E2)

Noise filtering is fundamental in the image processing for fluorescence microscopy, especially in scenarios where continuous and prolonged live-cell imaging is desired where the excitation power is maintained at a low level to minimize photo toxicity and photobleaching. The lower excitation power often results in a reduced signal-to-noise ratio. Traditional noise filtering is performed with a spatial filter, where each image for every given time instance is spatially filtered independently. However, because noise filtering in the spatial spectrum domain is equivalent to a convolution operation of the image with the kernel corresponds to the inverse Fourier transform of the low-pass filter, it is conceivable that the spatial-noise filtering would reduce the spatial resolution. On the other hand, to achieve a super-resolution movie, we are focusing on the sample conditions where the semi-static assumption is valid, which requires slow dynamics in the sample, and temporal-noise filtering has been proven useful (11). This is because slow dynamics ensures that the signal of interest exists in the low-frequency domain of the time axis while the noise is populated in the high-frequency domain in the time axis, therefore temporal-spectrum filtering can be effective. Additionally, because this filtering is performed along the time axis, the spatial resolution is not directly influenced.

We have implemented such temporal-noise filtering in *PySOFI* as a function module filtering.py. It is useful when analyzing multiple tiff stacks corresponding to consecutive time blocks. In such a scenario, the feature is assumed to be semi-static within each individual time block, and the corresponding tiff stack is analyzed independently. We can perform the temporal-noise filtering on the results across all time blocks to further enhance the image quality.

For example, we can perform the temporal noise filtering on the sixth-order-moment images calculated from 20 blocks of tiff stacks (each contains 200 frames) using the following scripts:

# First, we define the list of tiff stacks that corresponds to 20 different time blocks of a movie:

filenum = 20

filepath = '../sampledata'

filenames = ['Block' + str(i+1) + '.tif' for i in range(filenum)]

# Second, we perform the sixth-order moment calculations for all the blocks:

dset = { }; m_set = { }

for filename in filenames:

dset[filename] = pysofi.PysofiData(filepath, filename)

m_set[filename] = dset[filename].moment_image(order=6, finterp=False)

# Third, we generate a noise filter as a 1-dimensional Guasisan profile:

nf = masks.gauss1d_mask(shape =(1,21), sigma = 2)

# Last, we perform time-axis noise filtering:

m_filtered_set = filtering.noise_filter1d(dset, m_set, nf, return_option=True, return_type='dict')

The results from the temporal-noise filtering are stored as a dictionary in the m_filtered_set, where keys for elements are file names for each block of tiff images and values are the corresponding filtered images. The filtered images are also updated to each PysofiData objects as a PysofiData.filtered attribute. More detailed demonstrations are available in the corresponding Jupyter Notebook (E2).

### *ldrc* (E4)

One of the key challenges for high-order SOFI-cumulant calculations is the high dynamic range (HDR) of pixel intensities (1). The HDR issue also exists in the high-order-moment images (11). The *ldrc* (11) method was developed to mitigate such an issue (and is implemented in PySOFI) by re-scaling the pixel intensities of a given image based on a reference image. First, a reference image with the same feature but a more confined pixel intensity dynamic range is defined (e.g., the time average of the image series, or the second-order-moment or cumulant SOFI image). The compression is performed locally in a small window that scans across the image with a stride of 1 pixel. In each window, the pixel intensities of the original image are linearly re-scaled to share the same dynamic range as the reference window (11). The final value of each pixel is the average of the corresponding re-scaled values of them across all windows covering it.

In *PySOFI*, *ldrc* is implemented in the function module *ldrc.py* and integrated in the PysofiData.ldrc() method. The following scripts will calculate the sixth-order moment (*m6*) and the average image (*mean*) and perform *ldrc* on *m6* using *mean* as the reference:

# First, import the two relevant function modules, reconstruction and ldrc:

from pysofi import reconstruction as r

from pysofi import ldrc

# Define the path and file name of the data file:

filepath = '../sampledata'

filename = 'Block1.tif'

# Calculate the sixth-order moment (m6) and the average image (mean) using the reconstruction module:

m6 = r.calc_moment_im(filepath, filename, order=6, frames=[0, 50])

mean = r.average_image(filepath, filename)

# Compress the dynamic range of m6 with reference to mean using ldrc:

ldrc_im = ldrc.ldrc(mask_im=mean, input_im=m6, order=6, window_size=[20, 20])

We can also perform the *ldrc* processing directly through the PysofiData.ldrc() method using the following script:

# Load data into PysofiData object

filepath = '../sampledata'

filename = 'Block1.tif'

# Load the data into a PysofiData class object:

d = pysofi.PysofiData(filepath, filename)

# Calculate moments:

d.moment_image(order=6, finterp=False)

# Perfrom ldrc:

d.ldrc(mask_im=d.ave, input_im=d.moments_set[6], order=6, window_size=[20, 20])

**Figure 2:**
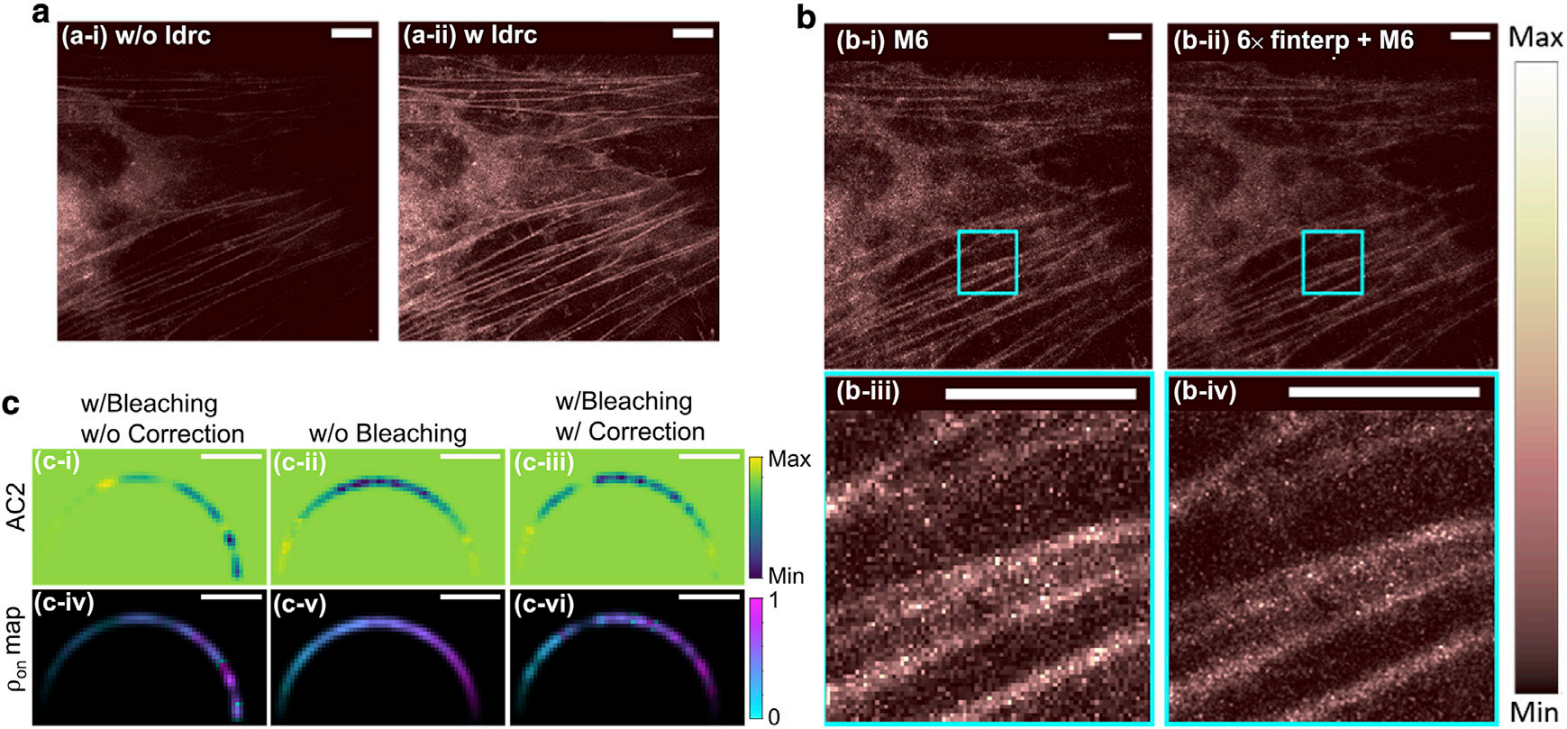
SOFI analysis demonstrations using *PySOFI*. (*A*) The experimental demonstration of *ldrc* algorithm on HeLa cells transfected with Dronpa-C12 fused to β-actin. Both images were processed using sixth-order moment, noise filtering, and deconvolution and were obtained during the SOFI 2.0 analysis pipeline, before (*a-i*) and after (*a-ii*) the *ldrc* step. Scale bars: 8 μm. (*B*) Experimental demonstration of FI algorithm on HeLa cells transfected with Dronpa-C12 fused to β-actin. (*b-i*) The sixth-order-moment-reconstructed image of the original wide-field acquisition. (*b-ii*) The sixth-order-moment image after the FI. *ldrc* is performed on both (*b-i*) and (*b-ii*) to compress the dynamic range of the reconstruction. (*b-iii*) A zoom-in box of (*b-i*). (*b-iv*) A zoom-in box of (*b-ii*). Scale bars, 8 μm. (*C*) The bleaching correction demonstration on a simulation data. The fourth-order cumulant image (*c-i* to *c-iii*) and multi-order cumulant analysis (*c-iv* to *c-vi*) is performed on a simulated video. A semicircle is populated with emitters with on-time ratios ranging from 0.01 (*left*) to 0.99 (*right*) with around 0.02 intervals. For emitters with photobleaching but without a BC step, the reconstructed pixel intensities (*c-i*) and emitters on-time ratio estimation (*c-iv*) are far off from the true values (*c-ii* and *c-v*), while the BC restores the information (*c-iii* and *c-vi*). Scale bars, 1.4 μm.

Note that the direct *ldrc* processing on *m6* often yields noisy results (we have demonstrated the results in the relevant Jupyter Notebook [E4]). However, *ldrc* plays an important role in the SOFI 2.0 pipeline, where the noise filtering and deconvolution are performed. In Fig. 2, we compare the partially processed SOFI 2.0 image (excluding *ldrc*) and the fully processed SOFI 2.0 image (including *ldrc*). We can see that the feature in the image is preserved without *ldrc* but is imperceptible due to the HDR issue. On the other hand, *ldrc* mitigates the HDR issue and provides an image where the dim features are shown more clearly. More detailed demonstrations are available in the corresponding Jupyter Notebook (E4).

### FI (E5)

fSOFI solves the finite pixilation problem of SOFI by adding virtual pixels using Fourier transforms (9). We have implemented the FI method in *PySOFI* to integrate the fSOFI analysis as an optional processing step. In our implementation, for the forward Fourier transform, the Fourier-transformation matrix was created with a size the same as the input image. We created the inverse Fourier-transformation matrix to include the extra interpolation position coordinates and omitted the “zero-padding” step in the Fourier space to avoid burdening the computation. With the FI, the input image/video is “projected” onto a more refined grid with finer pixel size.

In *PySOFI*, FI is implemented in the function module *finterp.py* and integrated in the PysofiData.finterp_tiffstack() method. We can perform the FI and save the output as a series of .tiff stacks. For example, the following scripts will calculate the two- and four-fold FI of the initial 100 frames from the example data set block10.tiff and save the interpolated images into two .tiff stacks: block10_InterpNum2.tiff and block10_InterpNum4.tiff, respectively.

# Import the relevant tools:

from pysofi import pysofi

# Load data into PysofiData object:

filepath = '../sampledata'

filename = 'Block10.tif'

d = pysofi.PysofiData(filepath, filename)

# Calculate the FI:

d.finterp_tiffstack(interp_num_lst=[2,4], frames=[0,100], save_option=True, return_option=False)

We can also perform the FI by using the finterp.py module as shown below:

# Import the relevant tools:

import tifffile as tiff

from functions import finterp

# Load a single image from the relevant data file:

filepath = '../sampledata'

filename = 'Block10.tif'

im = tiff.imread(filepath + '/' + filename, key=15) # read a frame

# Perform FI:

finterp_im2 = finterp.fourier_interp_array(im, [10]) # perform a 10-fold interpolation in the image.

Fig. 2 demonstrates the performance of the FI. Based on the Nyquist-Shannon sampling theorem (35,36), we recommend setting the interpolation factor at least two times the highest order for moment/cumulant reconstructions. For instance, if we plan to start the SOFI 2.0 pipeline with the sixth-order-moment image, we should pass interp_num_lst = [12], to d.finterp_tiffstack. However, in practice, depending on the dimension and length of the input file, FI might consume large processing memory and time. If computation resources are limited, we recommend saving the interpolated image stack as tiff files first instead of returning them and then processing the new file. Besides d.finterp_tiffstack, another option to include FI in the SOFI processing pipeline is to pass (finterp = True) and a interpolation factor (interp_num=6) when calculating the moment/cumulant reconstructions (see the *ldrc* (E4) section).

More detailed demonstrations are available in the corresponding Jupyter Notebook (E5).

### BC (E6)

Photobleaching of fluorescent probes is a general concern for super-resolution imaging-analysis methods. As for SOFI, photobleaching can cause errors in virtual brightness displayed in moment or cumulant images (26). Photobleaching leads to the loss of the fluorescence signal, which is mathematically equivalent as if the fluorophore is switched to a prolonged off state, degrading the quality of SOFI results. Therefore, BC is critical.

*PySOFI* employs a BC technique (11) that divides the whole video into shorter blocks based on the total signal intensity, I(t), where *t* is the time index and I(t) is the summation of all the pixel values of the image at time index *t*. The individual blocks are processed independently and combined subsequently to form a SOFI movie. First, the time series of the total signal intensity is smoothened to obtain a monotonically decreasing curve as an estimation of the bleaching profile of the movie. Then, based on the signal evolution over time, the sizes of the shorter blocks are determined so that the fractional signal decreases within each block (characterized by the BC factor, $f_{bc}$) is identical (11). The final SOFI moment/cumulant images with BC are the average of those calculated from individual blocks. Fig. 2 *C* shows that with the help of BC, the virtual brightness distribution and the photophysical properties (*c-iii*, *c-vi*) are successfully restored, yielding similar values as compared to the simulated case without bleaching (*c-ii*).

*PySOFI* offers two ways for BC. One way is through the PysofiData class as shown below:

# Import the relevant tools:

from pysofi import pysofi

# Load data into PysofiData object:

filepath = '../sampledata'

filename_bleach = 'Bleach_SlowVaryingRho_frame2000_Emi51.tif'

# Load the data set with bleaching into a PysofiData class object d_bleach:

d_bleach = pysofi.PysofiData(filepath, filename_bleach)

# Calculate the SOFI cumulants with bleach correction:

k_set_bleach_corrected = d_bleach.cumulants_images(highest_order=7, bleach_correction=True,

smooth_kernel=251, fbc=0.04)

We can also directly import the relevant function module reconstruction.py and perform BC as shown below:

# Import the function module and define the path and file name of the data set:

from pysofi import reconstruction as r

filepath = '../sampledata/simulations'

filename_bleach = 'Bleach_SlowVaryingRho_frame2000_Emi51.tif'

# Perform BC on the designated data set:

r.correct_bleaching(filepath, filename_bleach, fbc=0.04, smooth_kernel=251,

save_option=True, return_option=False)

In this example, we applied BC to a tiff stack, and the BC movie was saved as a separate tiff stack with the string “_bc” appended to the original file name.

More detailed demonstrations are available in the corresponding Jupyter Notebook (E6).

### Result visualization (E8)

We provide some simple visualization options in *PySOFI* to display either single or multiple images, with the option to adjust image contrast, and to display the image with a transparency map defined as an input parameter. Bokeh is used to offer an interactive display. More detailed demonstrations are available in the corresponding Jupyter Notebook (E8).

## Data availability

The data for this project is partially available on the project repository and are all organized on figshare(34). The usages of the example data sets are described below. Block1.tif to Block20.tif are live-cell imaging data (11) using HeLa cells labeled with Dronpa-C12 fused with β-actin. All the rest of the data sets are simulation data sets used and described in the example Jupyter Notebooks.

## Discussion

In this work, we developed *PySOFI*, an open source Python package for SOFI analyses. *PySOFI* contains the essential functionalities for conventional SOFI analysis as well as several derivative methods (9, 10, 11,26,10).

*PySOFI* adopts a simple architecture where all data-processing steps are implemented as independent function modules, and only one class module (the data class PysofiData) is used to manage the data-processing workflow.

The functions can be tested independently and used in different processing pipelines. A fast prototype on new analysis can be achieved by disseminating and reorganizing the processing step. One can implement additional processing steps as independent Python functions with the help of existing *PySOFI* functions. New functions can be used as standalone modules or can be integrated into the PysofiData class to support the new analysis pipeline. New classes can be constructed for different analysis pipelines as well.

We adopted Sphinx to manage the *PySOFI* documentation, which is available as an online documentation(33) to facilitate community usage. Additionally, each processing element of the analyzing pipeline is demonstrated in individual Jupyter Notebooks. In each notebook, we also provide instructions on how to tune processing variables and explore input data.

*PySOFI* is housed on GitHub(32) as an open-source repository, and any interested individuals can learn, inspect, validate, and contribute to the package. The user interactions on GitHub (e.g., fork, create pull requests, and report issues) engage community communications. We expect *PySOFI* to benefit general SOFI users for existing SOFI analysis as well as developers and new investigators interested in developing new SOFI-relevant analysis methods.

## Author contributions

Y.M. designed the *PySOFI* architecture, implemented the *PySOFI* package, and generated simulation videos for Fig. 2. X.Y. designed the research, supervised the development of the *PySOFI* package, developed the multi-order cumulant analysis algorithm, and collected experimental and simulation data. All authors analyzed the data, discussed the results, and wrote the manuscript.

## Declaration of interests

The authors declare no competing interests.
